# Levels of Proangiogenic Molecules and Terminal Complement Complex C5b-9 in the Crown of Circulating sEVs in Patients with Recurrent Glioblastomas: Relationship with Tumor Molecular Characteristics

**DOI:** 10.3390/cimb47020132

**Published:** 2025-02-18

**Authors:** Natalia Yunusova, Eldar Tulendinov, Dmitry Svarovsky, Anastasia Ryabova, Irina Kondakova, Anastasia Ponomaryova, Sergey Vtorushin, Stanislav Tabakaev, Dmitry Korshunov, Tatiana Shtam, Svetlana Tamkovich, Evgeny Choynzonov

**Affiliations:** 1 Cancer Research Institute, Tomsk National Research Medical Center, Russian Academy of Sciences, Kooperativny Str., 5, 634009 Tomsk, Russia; svarovsky.d.a@gmail.com (D.S.); ranigor@mail.ru (A.R.); anastasia-ponomaryova@rambler.ru (A.P.); wtorushin@rambler.ru (S.V.); stas_tab@bk.ru (S.T.);; 2 Department of Biochemistry and Molecular Biology with the Course of Clinical Laboratory Diagnostics, Siberian State Medical University (Siberian State Medical University of the Ministry of Health of the Russian Federation), Moskovsky Tract, 2, 634050 Tomsk, Russia; tylen-e01@mail.ru; 3 St. Petersburg Nuclear Physics Institute Named by B.P. Konstantinov of National Research Centre “Kurchatov Institute”, Orlova Roshcha 1, 188300 Gatchina, Russia; tatyana_shtam@mail.ru; 4 Department of Clinical Biochemistry, Novosibirsk State University, 2, Pirogov St., 630090 Novosibirsk, Russia; s.tamkovich@g.nsu.ru

**Keywords:** glioblastoma recurrence, extracellular vesicles, *MGMT* gene promoter methylation, p53, GFAP, terminal complement complex, VEGF-A, matrix metalloproteinases

## Abstract

Circulating small extracellular vesicles (sEVs) are emerging as potential biomarkers for glioblastoma progression. This study aimed to compare the levels of matrix metalloproteinases (MMP2 and MMP9), terminal complement complex (C5b-9), and VEGF-A in circulating sEVs in glioblastoma patients (GBMPs) with and without tumor recurrence. Using differential ultracentrifugation, sEVs were isolated from blood samples of GBMPs with no tumor recurrence for over one year (n = 6) and after first relapse (n = 14). The vesicles were characterized and quantified using flow cytometry. In both groups, C5b-9 was predominantly detected on tumor-specific circulating sEVs (glial fibrillary acidic protein (GFAP)-positive sEVs) with high VEGF-A expression, while C5b-9 was significantly less frequent on sEVs with low VEGF-A expression (*p* < 0.05). GFAP+VEGF+dimMMP2-C5b-9+ vesicles were rarely detected in GBMPs without relapse, suggesting their potential utility as biomarkers for a favorable relapse-free prognosis. In recurrent GBMPs, a positive correlation was observed between GFAP+VEGF+bright MMP2+C5b-9+ sEVs and *MGMT* gene promoter methylation levels (r = 0.543, *p* < 0.05). Additionally, a trend toward a negative correlation was found between GFAP+VEGF+bright MMP2+C5b-9- sEVs and mutant p53 expression in primary tumor tissue (r = −0.44, *p* = 0.114). These findings suggest that sEV profiles may serve as valuable prognostic markers for glioblastoma recurrence and treatment responses.

## 1. Introduction

The therapeutic efficacy of glioblastoma multiforme (GBM) treatment with radiotherapy/temozolomide is limited by increased invasiveness mediated by surviving cancer cells. In order to modulate the extracellular environment, malignant cells release various biopolymers (proteins and nucleic acids, etc.) into the blood and other physiological fluids, both as single molecules and as part of extracellular vesicles (EVs). Small extracellular vesicles (sEVs) are vesicles (40–200 nm) with lipid bilayer membrane carrying parental cell biopolymers, including specific tumor markers [1]. It has been shown that sEVs are able to penetrate both the damaged and intact blood–brain barrier [2]. Moreover, in vivo permeability assays revealed that hypoxic GBM-derived exosomes remained functional in blood circulation and induced the permeability of the blood–brain barrier. These cancer exosomes contain over-expressed VEGF-A, which enhances the permeability of the blood–brain barrier in an in vitro model by interrupting the expression of claudin-5 [3,4].

It is known that in GBM patients (GBMPs), unlike patients with other types of malignant tumors of the central nervous system, the concentration of one of the cytoskeleton proteins—glial fibrillary acidic protein (GFAP)—is increased in the blood [5]. In addition, this protein is presented on the surface of sEVs of plasma GBPs [6]. Other glioblastoma biomarkers such as the mutant variant of epidermal growth factor receptor EGFRvIII, podoplanin, and isocitrate dehydrogenase (IDH) have also been detected in sEVs [2]. In addition, GBM-secreted sEVs contain the matrix metalloproteinases (MMPs) MMP-2 and MMP-9 in their crown, which promote extensive hydrolysis of the extracellular matrix and increase tumor aggressiveness [7,8]. It has also been shown that the protein crown of sEVs is a platform for the activation of complement components, and that complement-dependent cytotoxicity is realized upon sorption of such vesicles on cells of various types, which may be involved in the mechanisms of degenerative and autoimmune diseases, tumor progression, and also explains some aspects of resistance to antitumor drugs [9,10,11,12].

The O6-methylguanine-DNA methyltransferase (*MGMT*) gene methylation status is crucial for the treatment stratification of GBMPs using one of the main drugs—temozolomide [13,14]. Other important molecular characteristics of the glioblastoma are Ki-67 expression [15] and the mutation profile of TP53 [16].

Although it is generally recognized that sEVs may be useful for diagnosing recurrence and monitoring the efficacy of antitumor therapy [2,17,18,19], there are no data in the literature on the association of cancer markers within sEVs with the molecular profile of primary glioblastoma.

In the present study, we examined the expression of MMP-2, MMP-9, terminal compliment complex (C5b-9), and VEGF-A in the crown of circulating sEVs in patients with recurrent and recurrence-free GBM in correlation with the molecular portrait of the primary tumor to identify a combination of promising markers for use in cancer diagnosis and prognosis using liquid biopsy.

## 2. Materials and Methods

### 2.1. Patients and Treatment

The study used the venous blood of GBMPs IDH-wild-type WHO CNS Grade 4, without signs of relapse for more than 1 year after completion of combined treatment (group without relapse, n = 6) and GBMPs after the 1st relapse (group with tumor relapse, n = 14). The age of GBMPs was 51.5 (45; 59) years in the first group and 45 (37; 55) years in the second group. Patients from both groups underwent microsurgical removal of the tumor followed by a course of chemoradiation therapy with temozolomide (Stupp protocol). In patients with the 1st relapse of glioblastoma, relapse developed within 3 to 20 months after surgery, with a median time of 7 months. After completion of chemoradiotherapy, patients received a median of 3 courses of adjuvant chemotherapy (range, 0 to 17 courses) with temozolomide until disease progression.

Blood sampling in the group of patients with tumor relapse was performed immediately after diagnosis of recurrence, but before the start of second-line chemotherapy according to the scheme: Bevacizumab 5–10 mg/kg on days 1 and 15 plus irinotecan 125–200 mg/m^2^ on days 1 and 15; cycle 28 days. Second-line chemotherapy was continued until the progression or occurrence of intolerable toxicity. Control examination during second-line chemotherapy was performed after every 2–3 courses of polychemotherapy. Recurrent disease was diagnosed based on the RANO HGG criteria [19].

Blood sampling from patients with a relapse-free course was carried out at least one year after completion of combined treatment in the absence of signs of relapse. Blood samples from patients without tumor relapse were collected within 22 to 77 months after surgery, with a median of 32 months. Patients received a median of 8 courses of adjuvant chemotherapy (range, 6 to 18 courses) with temozolomide. Adjuvant chemotherapy was interrupted due to metabolic remission detected by PET/CT with ^11^C-methionine or ^18^F-fluoroethyltyrosine.

Control MRI examinations of the brain were performed at the Cancer Research Institute using the MAGNETOM ESSENZA scanner (SIEMENS, Erlagen, Germany) with a magnetic field strength of 1.5 T in standard pulse sequences (in T1, T2, and FLAIR modes in the sagittal, axial and coronal planes, DWI (diffusion-weighted images), and ADC (apparent diffusion coefficient), slice thickness of 2.0–3.0 mm, d-factor of 1.0–3.0 mm) with mandatory acquisition of post-contrast images in T1 mode. MRI brain images were processed using the Makhaon Workstation program (Makhaon Doctor’s Workstation software, version 3.5.0.480 x64, Moscow, Russia). The T1 sequence after contrast medium infusion in the axial plane was used to visualize a lesions. By creating closed ROI polygons that outlined the lesion, the area of the contrast-enhanced tumor was determined in each successive axial section in which it was visualized. If the lesion was ring-shaped (in the axial plane) and was located around the postoperative cavity filled with cerebrospinal fluid, the closed polygon had the form of a ring with an external and internal contour. The lesion volume in cm^3^ was then determined using the “volume calculation” function embedded in the Makhaon Workstation software. In the group of patients without relapse, the residual volume was not estimated. If the “pseudoprogression” phenomenon was suspected, PET/CT with ^18^F-fluoroethyltyrosine or SPECT/CT with [^99m^Tc] Tc-1-thio-d-glucose were performed.

Tumor progression patterns were classified as local progression: recurrence adjacent to the resection cavity or original tumor site; distant progression: recurrence not adjacent to the resection cavity or original tumor site; subependymal spread: lesions disseminated along with the subependymal zone; leptomeningeal spread: leptomeningeal contrast enhancement around the contours of the gyri and sulci or multiple nodular deposited in the subarachnoid space [20,21]. Distant, subependymal, and leptomeningeal types of progression were considered as non-local.

The exclusion criteria were as follows: contraindications to surgical treatment of the primary tumor; inconsistency of morphological and molecular genetic characteristics of the tumor with the diagnosis of IDH-wild-type glioblastoma in accordance with the 2021 WHO classification of CNS tumors; the combination treatment protocol did not include the Stupp protocol; contraindications to second-line chemotherapy; and the failure to monitor patients and patients’ refusal to participate in the study.

The study complied with the standards of the Helsinki Declaration and was approved by the independent ethics committee of the Cancer Research Institute of the Tomsk National Research Medical Center of the Russian Academy of Sciences (protocol No. 9, 24 September 2021). All patients included in the study signed the informed consent.

### 2.2. Isolation and Characterization of sEVs

Furthermore, sEVs were isolated by ultrafiltration followed by ultracentrifugation. Briefly, venous blood (27 mL) was collected in vacutainers with K_3_EDTA. Cell fractionation was performed at 4 °C for 20 min at 1000× *g*. Plasma from the first centrifugation was collected into a new tube and centrifuged for 15 min at 14,000× *g* and 4 °C. The resulting supernatant was diluted with PBS to a total volume of 33 mL, and the samples were filtered through filters with a pore diameter of 220 nm (PES, Wuxi NEST Biotechnology Co, Ltd., Wuxi, China). The filtrate was subjected to subsequent ultracentrifugation in an ultracentrifuge with a bucket rotor (Optima XPN 80, Beckman Coulter, Brea, CA, USA) at 4 °C for 90 min. The resulting pellet was resuspended in PBS and centrifuged once more under the same conditions. The sEV aliquots were frozen in liquid nitrogen and stored at −80 °C.

The distribution and concentration of the isolated vesicles were estimated by the nanoparticle tracking analysis (NTA) using a NanoSight^®^ LM10 device (Malvern Instruments, Worcestershire, UK). To optimize the measurements, sEV samples were diluted with PBS at proportions of 1:100, 1:1000, and 1:10,000, and then the data were analyzed. The average vesicle size, their size distribution, and vesicle concentration were investigated by one-minute analysis.

For transmission electron microscopy (TEM), the sEVs were absorbed on the copper grid covered with formvar film, stabilized by carbon, and then the grids were exposed with 2% phosphotungstic acid. The preparations were analyzed on a Talos L120C electron microscope (Thermoscientific, Waltham, MA, USA).

The protein level in isolated vesicles was estimated using the fluorimetric method, with modification. Briefly, 10 μL of vesicle preparations were mixed with 3 μL of lysis buffer (0.25 M Tris-HCl, 8% SDS, 0.2 M DTT, pH 6.8), incubated on ice for 10 min, then boiled at 95 °C for 10 min. After centrifugation at 12,000× *g* for 10 min, the whole supernatant was mixed with 3 μL of fluorescent solution (3 mg/mL, CAS 38183, BLD Pharm, Shanghai, China) in dimethyl sulfoxide. Measurements were performed on an Agilent BioTek Cytation 1 Cell Imaging Multimode Reader (Biotek, Shoreline, WA, USA), with an excitation wavelength of 365 nm and emission wavelength of 470 nm.

### 2.3. Analysis of CD9/CD63/CD81/CD24 Subpopulations on the sEV Crown Using Flow Cytometry

Five microliters of 4 μm-diameter aldehyde/sulfate latex beads (Molecular Probes, Eugene, OR, USA) were subjected to two washing steps using 100 µL 0.1 M MES buffer (pH 5.5) under centrifugation conditions (3000× *g*, 15 min, and room temperature). Following washing, the beads were resuspended in 25 µL MES buffer and subsequently incubated with 3 µg anti-CD9 (SAA0003, Antibody System, Schiltigheim, France) monoclonal antibodies at room temperature for 14 h, with gentle agitation. The aliquots of sEVs (about 30 μg vesicular protein) were incubated with 3 × 10^5^ antibody-coated latex beads in 150 μL of PBS at 4 °C for 14 h, under gentle agitation. The reaction was blocked with 0.2 M glycine for 30 min at 4 °C. The sEV–antibody–bead complexes were washed twice with washing buffer (2% EVs depleted bovine serum in PBS) and incubated with 5 µL anti-human Fc-receptor-binding inhibitor (Invitrogen, Elabscience, Houston, TX, USA) at room temperature for 10 min, with washing. For an analysis of CD9/CD63/CD81/CD24 subpopulations on the surface of CD9-positive sEVs, FITC-conjugated antibodies against tetraspanins (CD63 and CD81) and antibodies against CD24 (BD Biosciences, Heidelberg, Germany, 5 µL per test) were added to sEV–antibody–bead complexes. After incubation for 20 min at room temperature, the complexes were then washed with washing buffer. Flow cytometry was performed on a cytometer Cytoflex (Becman Coulter, BioBay, Suzhou, China) and the data were analyzed using CytExpert 2.4 software (Becman Coulter, Brea, CA, USA). The median fluorescence intensity (MFI) of the EVs was analyzed in comparison with the isotypic and negative controls (BD Bioscience, Milpitas, CA, USA).

### 2.4. Analysis of MMP9/MMP2/VEGF-A/C5b-9 Subpopulations on the Surface of CD9-Positive and GFAP-Positive sEVs Using Flow Cytometry

The CD9 antibody-coated or GFAP (MA5-29255, Invitrogen, Shanghai, China, 3 µg for test) monoclonal antibody-coated aldehyde/sulfate latex bead–sEVs complexes were obtained as described above. Then, these complexes were washed twice with washing buffer and incubated with 5 µL anti-human Fc-receptor-binding inhibitor (Invitrogen, Elabscience, CA, USA) at room temperature for 10 min, with washing. The CD9 antibody-coated or GFAP antibody-coated aldehyde/sulfate latex bead–sEVs complexes were stained with antibodies MMP9-FITC (FAA553Hu81, Cloud-Clone Corp., Wuhan, China, 2 μL per test), C5b-9-APC (FHN10210-APC, AntibodySystem, Schiltigheim, France, 0.5 μL per test), MMP2-PE (FAA100Hu41, Cloud-Clone Corp., China, 2 μL per test), and VEGFA-PerCP/C5.5 (FAA143Huj2, Cloud-Clone Corp., China, 2 μL per test) for 20 min. Then, the complexes were washed with washing buffer. Flow cytometry was performed on a cytometer Cytoflex (Becman Coulter, BioBay, China) and data were analyzed with CytExpert 2 software (Becman Coulter, CA, USA). Initially, the “Beads” were gated with sorbed EVs. In the gated “Beads”, 10,000 events were analyzed in each sample at a high flow rate. Depending on the expression of VEGF-A on EVs, the VEGF+dim and VEGF+bright subpopulations were gated. We analyzed data on the composition of CD9+EVs (total EVs fraction) and GFAP+EVs (glioblastoma-specific EVs) separately.

### 2.5. Immunohistochemistry (IHC)

Surgically resected tumor samples were collected from patients diagnosed with glioma. The samples were fixed in 10% neutral buffered formalin and embedded in paraffin according to standard histopathological procedures. Representative tumor regions were selected by a pathologist for further analysis.

Tissue sections that were 3–4 μm thick were prepared from formalin-fixed paraffin blocks of glioma. Immunohistochemical staining was performed using the BOND-MAX Fully Automated IHC and ISH Staining System (Leica Biosystems, Newcastle, UK). The primary antibodies against p53 (clone DO-7, RTU, Leica Biosystems, Newcastle, UK) and Ki-67 (clone SP6, 1:400, Cell Marque, Rocklin CA, USA) were used. The Polymer Refine Detection System (Leica Biosystems, Newcastle, UK) was used for detection. Tissue sections mounted on microscope slides were deparaffinized in xylene and rehydrated through a graded series of ethanol solutions. Antigen retrieval was performed using thermal treatment in citrate buffer, pH 6, after which the slides were placed into an automated Leica BOND MAX staining system. All staining steps, including primary antibody incubation and detection, were fully automated and performed according to the manufacturer’s standard protocol.

The evaluation of p53 mutation status is based on the nuclear expression of the p53 protein. A semi-quantitative scoring system is used for analysis: 0: No staining, 1+: Weak expression (fewer than 10% of tumor cell nuclei stained), 2+: Moderate expression (10–50% of tumor cell nuclei stained), and 3+: Strong expression (more than 50% of tumor cell nuclei stained). Mutant-type p53 is identified by strong nuclear expression (2+ or 3+). Wild-type p53 is indicated by weak or absent expression (0 or 1+).

Ki-67 proliferative activity was assessed based solely on nuclear staining. The percentage of Ki-67-positive nuclei in tumor cells was calculated. Then, 3–5 fields of view with the highest staining intensity (so-called “hot spots”) were analyzed under ×400 magnification. For each field of view, at least 500 tumor cell nuclei were counted, and the number of Ki-67-positive nuclei was recorded. The Ki-67 proliferation index is calculated as the percentage of Ki-67-positive nuclei out of the total number of nuclei (number of positive nuclei/total number of nuclei × 100%).

### 2.6. DNA Isolation, Bisulfite Conversion, and Methyl-Specific TaqMan PCR (MSP)

DNA was isolated from formalin-fixed, paraffin-embedded tissues using the QIAmp DNA Micro kit (Qiagen, Venlo, The Netherlands) and modified with sodium bisulfite followed by purification using «EZ DNA Methylation-Direct Kit» (Zymo Research, Orange, CA, USA). The converted DNA was used for the following analysis of the MGMT methylation status.

The concentration of the methylated form of the *MGMT* gene was assessed using quantitative MSP with fluorescence dye EvaGreen (Biotium, San Francisco, CA, USA). Each reaction was carried out in a final volume of 20 µL containing 2 µL of DNA, 60 nM of a specific primer for each gene, 1 U of Taq polymerase, 10× PCR buffer and 25 nM dNTP, and 50 nM MgCl_2_. The PCR program included the following steps: heating at 95 °C for 10 min, then amplification for 40 cycles in the following mode: 95 °C for 30 s, 59 °C for 30 s, 72 °C for 20 s, and detection of fluorescence signal at 77.5 °C for 15 s. Amplifications were carried out in the CFX96 instrument (Bio-Rad, Berkeley, CA, USA). Concentrations were calculated using the calibration curve made by serial dilutions of the standard—completely methylated bisulfite-converted human DNA with a known concentration (Zymo Research, Orange, CA, USA) (100%–80%–25%–13%–6%–3%–1%) [22].

The work was carried out using the equipment of the shared use center of the Tomsk Research Medical Center of the Russian Academy of Sciences “Medical Genomics”, the Laboratory of experimental biochemistry and biology of the Siberian State Medical University, Tomsk (Head—D.Sci., MD, Spirina L.V.) and the Nanotech shared use center of the Institute of Strength Physics and Materials Science of the Siberian Branch of the Russian Academy of Sciences (Tomsk).

### 2.7. Statistical Analysis

Statistical analysis was carried out using Statistica 10 (TIBCO Software, Palo Alto, CA, USA) software. All results are presented as medians with interquartile range, Me (Q1; Q3). Mann–Whitney tests were used to evaluate statistical differences between groups, and *p*-values < 0.05 were considered statistically significant. Correlation analysis of the data was carried out with the Spearman Rank Correlation test. Furthermore, *p*-values < 0.05 were considered statistically significant.

## 3. Results

**Figure 1:**
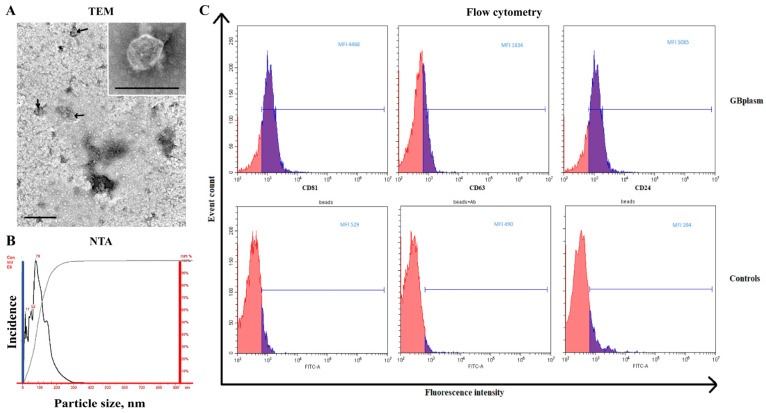
Identification of isolated sEVs. TEM of isolated EVSs. Electron microscopy, negative staining with phosphotungstic acid (×400). The scale bar is shown in the photo (**A**); size distribution of plasma sEVs isolated from the blood of MGPs, data of NTA (**B**); (**C**) expression of CD63, CD81, and CD24 on CD9-positive sEVs of blood plasma of MGPs. Representative median fluorescence intensity (MFI) values are shown for flow cytometry. Each study was performed in triplicate. For isotype controls (bottom row, histogram at right), labeled CD9 bead–sEV complexes were incubated with mouse FITC IgG1, k Isotype control or mouse FITC IgG2a, or k Isotype control. One of the representative isotypic controls is shown. For the negative control, nothing was added to the CD9 antibody-labeled latex particles (bottom row, histogram at left) or incubated with FITC-labeled anti-human antibodies (anti-CD63, anti-CD81, or anti-CD24) without EVs. One of the representative negative controls is shown (bottom row, histogram in the center).

According to electron microscopy, sEVs were visualized as rounded cup-shaped membrane structures. The number of vesicles was sufficient for further studies (Figure 1A). Figure 1 also shows the NTA data and tetraspanin expression in the isolated sEVs. Circulating sEVs from GBMPs (group with tumor relapse) had a mean size of 88.9 nm, with a mode of 69.3 nm and a standard deviation of 37 nm, while EVs from non-relapsed GBMPs had the following characteristics: a mean size of 94.0 nm, with a mode of 68.0 nm and a standard deviation of 39.1 nm (*p* > 0.05). About 95% of the isolated vesicles were less than 200 nm in size (Figure 1B). The mean sEV concentration in samples from patients with and without relapses did not differ and was 4.7 ± 0.88 × 10^12^ particles/ml blood and 8.0 ± 2.8 × 10^12^ particles/mL blood, respectively. Typical tetraspanins CD9, CD81, CD63, and glycoprotein CD24 were expressed on the vesicles (Figure 1C).

**Figure 2:**
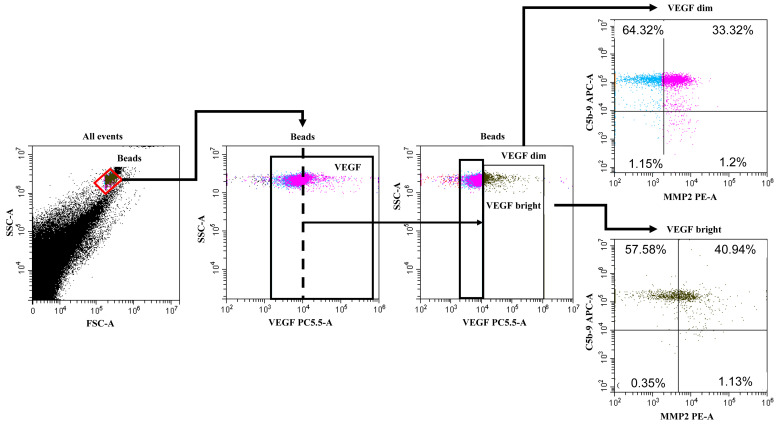
Gating strategy. Initially, a gate of latex particles with sorbed EVs was isolated, then a subpopulation of VEGF-positive EVs was identified, then VEGF-dim and VEGF-bright populations were isolated. Then, populations with different expressions of C5b-9 and MMP2 were detected in these subpopulations.

The gating strategy for vesicles stained with the MMP9/MMP2/VEGF-A/C5b-9 antibody complex is shown in Figure 2.

**Figure 3:**
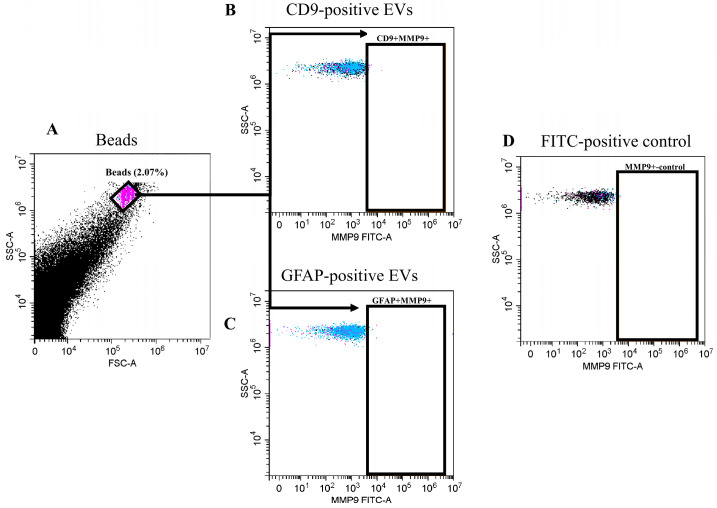
MMP9 expression in blood plasma EVs of GBMPs, flow cytometry. (**A**) Forward scatter area (FSC-A) versus side scatter area (SSC-A) dot plot representing sEV samples adsorbed on aldehyde–sulfate latex beads labeled with anti-CD9 or anti-GFAP antibodies, (**B**) MMP9-positive EVs within CD9-positive EVs in patients with glioblastoma, (**C**) MMP9-positive EVs within GFAP-positive EVs in patients with glioblastoma, and (**D**) single dye control of FITC-conjugated MMP9-antibody.

According to the flow cytometry data, MMP9 was not expressed on either CD9-positive or GFAP-positive EVs (Figure 3).

When analyzing the total fraction of sEVs (CD9-positive EVs), no statistically significant differences were found between the groups with recurrent and relapse-free glioblastoma (Table 1).

When analyzing the phenotypes of GFAP-positive EVs, statistical differences were found in the content of vesicles with the VEGFdim+MMP2+C5b-9- and VEGFdim+MMP2-C5b-9+ phenotype between patients with recurrent and non-recurrent GBM (*p* < 0.05) (Table 2).

In the groups both with and without relapse, C5b-9 was frequently detected on GFAP-positive (tumor-specific) circulating EVs with high VEGF-A expression, whereas C5b-9 was significantly less frequently detected on EVs with low VEGF-A expression (*p* < 0.05). GFAP+VEGF+dim MMP2-C5b-9+ vesicles were almost never found in GBMPs without relapse.

We also analyzed changes in the *MGMT* methylation level and Ki67 and p53 tumor protein levels in their dependence on clinical characteristics of GBMPs (age, sex, relapse status, progression pattern, and residual tumor volume according to the MRI data) (Table 3, Table 4 and Table 5).

It was revealed that the relapse status in patients was associated only with the level of Ki67 in the primary tumor tissue (*p* = 0.002). No other statistically significant associations of the *MGMT* gene promoter methylation level and Ki67 and p53 tumor protein levels with dependence on clinical characteristics of GBMPs (age, sex, relapse status, progression pattern, and residual tumor volume) were found.

**Figure 4:**
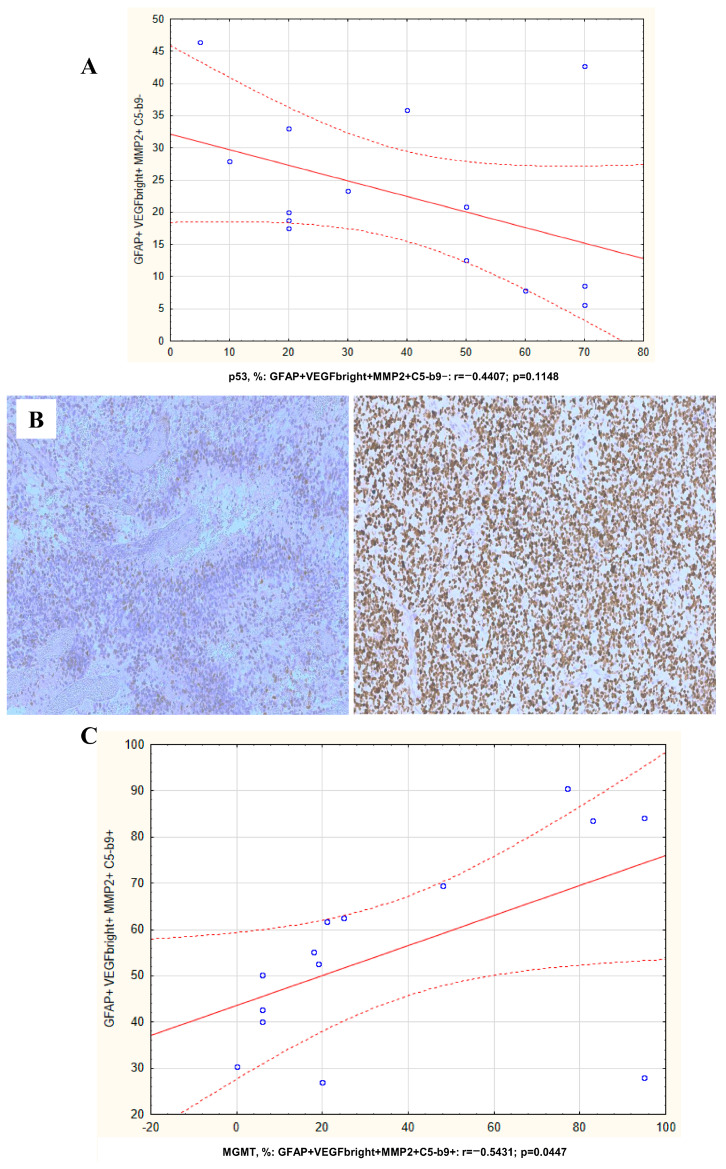
Correlation of plasma sEVs with molecular markers of primary tumor in GBMPs. (**A**) Correlation of GFAP+VEGF+bright MMP2+C5b-9- plasma sEVs levels with intratumoral expression of p53 protein; the photographs are from two representative GBMPs; (**B**) heterogenous and weak staining with antibody for p53 in tumor—«wild type» in the left photo. Strong and diffuse staining with antibody for p53 in the tumor—«mutant type» in the right photo. Immunohistochemistry (×200). Correlation of GFAP-positive plasma sEVs with phenotype GFAP+VEGF+bright MMP2+C5b-9+ in GBMPs with concentration of methylated fragments of the *MGMT* gene promoter in primary tumors (**C**).

In patients with recurrent glioblastoma, a tendency toward a negative correlation was found between the content of sEVs with the GFAP+VEGF+bright MMP2+C5b-9- phenotype and the expression of mutant p53 in the primary tumor tissue (r = −0.44, *p* = 0.114) (Figure 4A,B). A positive correlation was also found between the content of EVs with the GFAP+VEGF+bright MMP2+C5b-9+ phenotype and the *MGMT* gene promoter methylation level (r = 0.543, *p* = 0.044) (Figure 4C).

## 4. Discussion

Most of the data in the literature indicate the presence of MMP9 on the surface of EVs of various origins [23,24,25]. Our own data indicate the significant expression of MMP9 on the surface of sEVs and exosomes in patients with ovarian cancer and borderline ovarian tumors (plasma and ascites vesicles), as well as in patients with colon polyps and colorectal cancer [23,26]. However, in GBMPs, MMP9 was not detected on circulating EVs, which is an interesting feature and requires further study.

GFAP is a protein that is classically used to identify malignancies of glial origin, such as astrocytoma and glioblastoma [5]. GFAP-positive circulating EVs have previously been used to monitor anti-relapse therapy for GBMPs. Small CD9+/GFAP+/Survivin+ and CD9+/Survivin+ EVs are present in the circulation of GBMPs, and a sustained decrease in their numbers after anti-survivin immunotherapy may be associated with longer progression-free survival. Thus, the determination of GFAP-positive EVs in blood plasma could apparently be useful in monitoring GBMP therapy [17].

It is known that complement activation in the composition of IgG-loaded EVs in blood plasma in patients with diabetes mellitus leads to C5b-9 deposition in the retina and contributes to endothelial damage and the progression of diabetic retinopathy [9]. It has been previously shown that astrocyte-derived EVs and, less effectively, neuron-derived EVs in Alzheimer’s disease patients, induced C5b-9 expression on recipient neurons, membrane disruption, decreased neurite density, and decreased cell viability in rat cortical neurons and human iPSC-derived neurons. These effects were not induced by non-specific CD81+ EVs in Alzheimer’s disease patient plasma or by total plasma EVs from healthy donors [10]. Our studies have shown that in both groups, C5b-9 is detected quite frequently on GFAP-positive (tumor-specific) circulating EVs with high VEGF-A expression, whereas C5b-9 was significantly less frequently detected on GFAP-positive EVs with low VEGF-A expression. GFAP+VEGF+dim MMP2-C5b-9+ vesicles were almost never found in GBMPs without relapse (*p* < 0.05). Circulating EVs play an important role in the progression of glioblastomas; their effect can be associated with both the immunomodulatory effect and the specific properties of EVs. It has been shown that sEVs of a tumor origin can promote angiogenesis, decreasing the effect of bevacuzumab due to the presence of heparin-associated and bevacuzumab-uninhibited VEGF-A on their surface [27,28].

The revealed tendency toward a negative correlation between the content of circulating EVs with the GFAP+VEGF+bright MMP2+C5b-9- phenotype and the expression of mutant p53 in tumor tissue can be explained by several mechanisms associated with tumor biology and EV secretion. First, p53 plays a key role in the regulation of cellular secretion and the composition of EVs. Normally, it is involved in maintaining the homeostasis of the tumor microenvironment, including the expression of angiogenesis factors and extracellular matrix remodeling. Mutations in p53, characteristic of aggressive tumors, lead to the dysregulation of these processes, which can reduce the level of VEGF+bright MMP2+C5b-9- EVs in circulation. Such changes may reflect a decrease in the angiogenic properties of the tumor mediated by EVs [29]. Secondly, it is known that mutant p53 is able to alter the expression of genes regulating the secretion and composition of EVs. In particular, a decrease in the level of VEGF-A and MMP2 presented on the surface of EVs may be associated with a disruption of the functions of p53 as a tumor suppressor, which, in turn, affects matrix remodeling and the induction of angiogenesis. Third, aggressive tumors associated with p53 mutations can redistribute cellular energy and metabolic resources, reducing the secretion of EVs with angiogenic properties and focusing on the release of vesicles with other phenotypes that promote invasiveness, resistance to therapy, and immunomodulation [30].

At the same time, a positive correlation was found between the content of cytotoxic EVs with the GFAP+VEGF+bright MMP2+C5b-9+ phenotype and the methylation level of the *MGMT* gene promoter. The methylation of *MGMT* has been extensively studied as a prognosis biomarker in GBMPs. Its significance has been studied in various subgroups, including age, gender, and even race. It has been shown that *MGMT* methylation is significantly associated with progression-free and overall survival in GBMPs. Moreover, the methylation of *MGMT* is a valid biomarker for predicting responses to therapy with alkylating agents [2,22]. The presence of a correlation between the composition of circulating EVs and the MGMT promoter’s methylation level suggests the presence of common epigenetic regulation of these processes. It is known that p53 is able to influence MGMT expression, as well as epigenetic changes associated with tumor progression [31].

Analysis of exosome secretion from primary glial cultures using flow cytometry with antibodies against CD63, GFAP, and Tsg101 revealed an increased number of exosomes after stimulation of cells with IL-1β. This proves the feasibility of secreting “true” GFAP-positive EVs and detecting such vesicles via high-throughput flow cytometry [32]. As a rule, recurrent glioblastomas are characterized by diffuse pronounced staining for GFAP; therefore, GFAP-positive EVs can be present in the blood plasma in large quantities [17]. However, given the data on the high serum GFAP levels in such patients [5], circulating EVs with the GFAP+VEGF+bright MMP2+C5b-9+ phenotype can be considered as a mixed fraction of tumor-specific EVs and a subfraction of the total pool of plasma EVs, with accumulation of the four biomarkers GFAP, VEGF, MMP2, and C5b-9 on their surface. Further study is needed to determine the detailed composition of this fraction of circulating EVs.

This study has limitations. First of all, the small size of the cohort of GBMPs. We believe that expanding the cohort of patients and analyzing the composition of EVs in dynamics before and after second-line chemotherapy will allow us to obtain results that can be used for the personalized therapy of GBMPs.

## 5. Conclusions

In the groups both with and without relapse, C5b-9 was frequently detected on GFAP-positive (tumor-specific) circulating sEVs with high VEGF-A expression, whereas MAC was significantly less frequently detected on EVs with low VEGF-A expression (*p* < 0.05). GFAP+VEGF+dim MMP2-C5b-9+ vesicles were practically not detected in the blood of relapse-free patients, and the determination of these vesicles would be useful for a more accurate stratification of GBMPs with a relapse-free favorable course. In patients with relapsed glioblastoma, a tendency toward a negative correlation was found between the content of sEVs with the GFAP+VEGF+bright MMP2+C5b-9- phenotype and the expression of mutant p53 in the primary tumor tissue, which requires further study. Taking into account the found associations between the phenotype of circulating sEVs and the methylation levels of the *MGMT* gene promoter, it can be assumed that circulating cytotoxic vesicles with the GFAP+VEGF+bright MMP2+C5b-9+ phenotype could potentially be useful both for predicting overall and relapse-free survival and as an available predictive marker for planning therapy with alkylating agents.

## Figures and Tables

**Table 1:** Levels of MMPs, VEGF-A, and C5b-9 on CD9-positive EVs, Me (Q1; Q3).

EV Population, %	GBMPs, First Relapse, Before Treatment	GBMPs Without Relapse	*p*-Level
VEGF+dim MMP2+C5b-9+	32.0 (23.7; 37.9)	24.3 (3.36; 34.0)	0.490
VEGF+dim MMP2-C5b-9+	7.98 (5.42; 47.8)	2.91 (1.76; 7.30)	0.320
VEGF+dim MMP2-C5b-9-	10.8 (6.80; 21.9)	11.4 (10.7; 50.7)	0.550
VEGF+dim MMP2+C5b-9-	35.5 (4.67; 45.1)	43.0 (40.2; 47.9)	0.491
VEGF+bright MMP2+C5b-9+	61.6 (48.3; 71.1)	65.0 (62.8; 84.4)	0.692
VEGF+bright MMP2-C5b-9+	10.3 (1.38; 18.1)	8.33 (1.16; 10.2)	0.550
VEGF+bright MMP2-C5b-9-	3.78 (1.67; 12.3)	1.67 (0.58; 14.9)	0.660
VEGF+bright MMP2+C5b-9-	16.5 (7.08; 21.0)	13.9 (12.1; 15.5)	0.920

**Table 2:** Levels of MMPs, VEGF-A, and C5b-9 on GFAP-positive EVs, Me (Q1; Q3).

EV Population, %	GBMPs, First Relapse, Before Treatment	GBMPs Without Relapse	*p*-Level
VEGF+dim MMP2+C5b-9+	4.36 (2.28; 28.2)	8.17 (1.44; 16.6)	0.690
VEGF+dim MMP2-C5b-9+	6.19 (2.36; 7.13)	1.79 (1.79; 2.78)	0.040
VEGF+dim MMP2-C5b-9-	40.9 (16.4; 48.1)	22.8 (20.7; 27.3)	0.920
VEGF+dim MMP2+C5b-9-	43.7 (41.5; 49.6)	60.6 (58.7; 62.8)	0.045
VEGF+bright MMP2+C5b-9+	52.5 (40.0; 69.4)	64.5 (41.8; 68.4)	0.950
VEGF+bright MMP2-C5b-9+	4.00 (2.21; 5.41)	1.95 (1.45; 3.48)	0.180
VEGF+bright MMP2-C5b-9-	11.9 (4.63; 17.4)	7.47 (3.86; 8.70)	0.551
VEGF+bright MMP2+C5b-9-	20.4 (12.5; 33.1)	25.3 (23.1; 26.9)	0.490

**Table 3:** *MGMT* gene promotor methylation level and clinico-pathological characteristics of GBMPs, Me (Q1; Q3).

Parameters, n	*MGMT* Gene Promotor Methylation Level, %	*p*-Level
**Age (years)**		
≤52, n = 11	6.00 (1.00; 48.0)	0.734
>52, n = 9	19.0 (0–79.0)	
**Sex**		
Female, n = 7	12.0 (1.00; 83.0)	0.101
Male, n = 13	6.0 (0; 40.0)	
**Relapse status**		
First relapse, n = 14	18.1 (1.00; 48.0)	0.629
No relapse, n = 6	4.00 (0–40.0)	
**Progression pattern**		
Local, n = 10	19.5 (6.00; 62.5)	0.379
Non-local, n = 4	9.50 (0–51.0)	
**Residual tumor volume, cm^3^**		
≤22.6, n = 8	19.0 (6.00; 83.0)	0.351
>22.6, n = 6	6.00 (1.00; 25.0)	

**Table 4:** Tumor p53 protein level and clinico-pathological characteristics of GBMPs, Me (Q1; Q3).

Parameters, n	Tumor p53 Protein Level, %	*p*-Level
**Age (years)**		
≤52, n = 11	40.0 (30.0; 70.0)	0.292
>52, n = 9	20.0 (15.0–50.0)	
**Sex**		
Female, n = 7	50.0 (30.0; 70.0)	0.140
Male, n = 13	20.0 (15.0; 40.0)	
**Relapse status**		
First relapse, n = 14	30.0 (20.0; 50.0)	0.542
No relapse, n = 6	95.0 (2.00–100)	
**Progression pattern**		
Local, n = 10	30.0 (20.0; 50.0)	0.851
Non-local, n = 4	25.0 (15.0–45.0)	
**Residual tumor volume, cm^3^**		
≤22.6, n = 8	30.0 (20.0; 60.0)	0.254
>22.6, n = 6	20.0 (5.00; 40.0)	

**Table 5:** Tumor Ki67 protein level and clinico-pathological characteristics of GBMPs, Me (Q1; Q3).

Parameters, n	Tumor Ki67 Protein Level, %	*p*-Level
**Age (years)**		
≤52, n = 11	20.0 (8.00; 25.0)	0.386
>52, n = 9	10.0 (10.0–15.0)	
**Sex**		
Female, n = 7	19.0 (10.0; 20.0)	0.927
Male, n = 13	10.0 (10.0; 30.0)	
**Relapse status**		
First relapse, n = 14	20.0 (10.0; 25.0)	0.002
No relapse, n = 6	0.20 (0.15–0.30)	
**Progression pattern**		
Local, n = 10	19.0 (10.0; 22.5)	0.379
Non-local, n = 4	25.0 (15.0–30.0)	
**Residual tumor volume, cm^3^**		
≤22.6, n = 8	20.0 (10.0; 25.0)	0.351
>22.6, n = 6	15.0 (10.0; 20.0)	

## Data Availability

The data presented in this study are available upon request from the corresponding authors.
